# Supplementary material for: Optimizing resource allocation in Miscanthus breeding via sparse testing designs for genomic prediction
Source: Front Plant Sci. 2026 Aug 4;17:1834912. doi: 10.3389/fpls.2026.1834912 (PMC13481439; doi:10.3389/fpls.2026.1834912)
Supplement: Supplementary file 1 [file DataSheet1.pdf]

Supplementary Material

1.1 Supplementary Figures

Figure S1

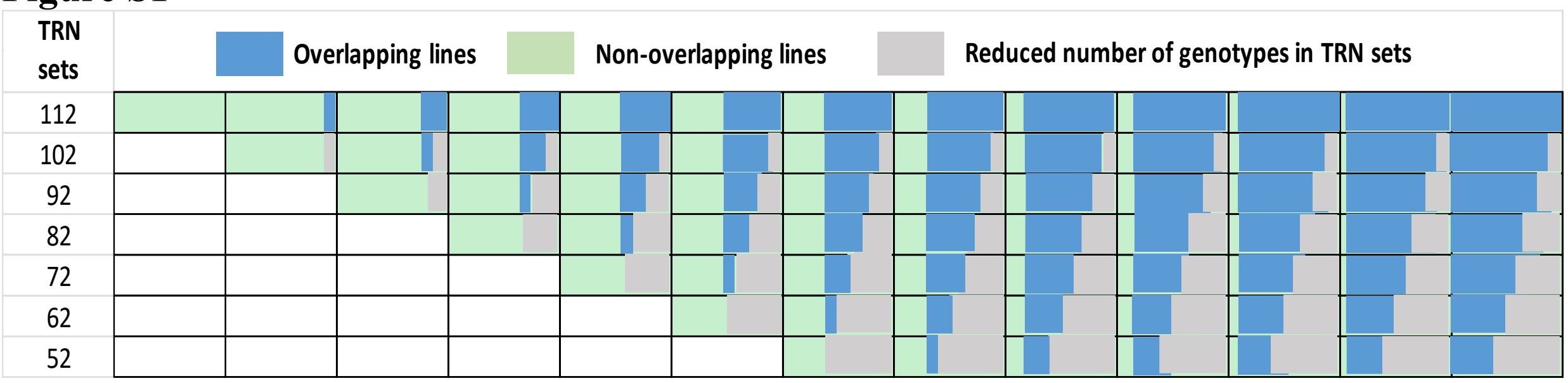

Graphical presentation of different allocation schemes. The rows present different TRAINING sets (from 112 until 52) and the columns present the allocation designs. The blue color shows the overlapped sets, light green presents the non-overlapping sets and the grey color displays the reduced TRAINING sets. Across designs (from left to right), the number of non-overlapping genotypes decreases while the number of overlapping genotypes increases.

Figures S2-S5

The PA between predicted and observed value for each trait within each environment.

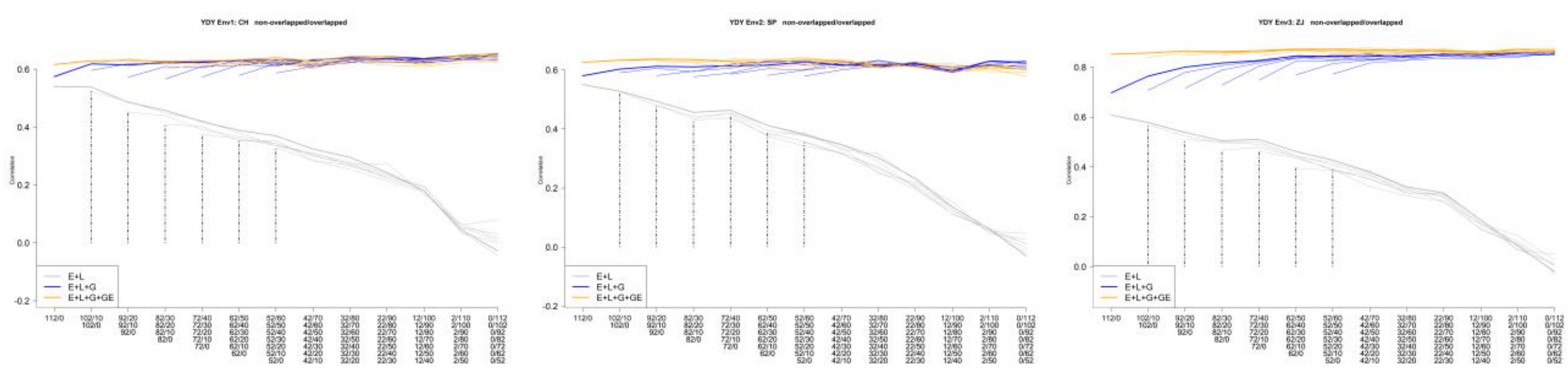

Figure S2 The correlation between predicted and observed values for varying TRAINING sets and allocation designs in YDY [Left: correlation for YDY in environment 1, Middle: correlation for YDY in environment 2, Right: correlation for YDY in environment 3] within each environment in three genomic prediction models, M1: **E+L;** M2: **E+L+G;** M3: **E+L+G+GE**. Bold lines show the average PA for the largest calibration set (112 genotypes). Dotted lines show the reduced training sets (102, 92, 82, 72, 62, and 52 genotypes). X axis shows the compositions of different non-overlapping (NO) / overlapping (O) allocation designs. Leftmost side of the X axis shows compositions for completely NO genotypes with 0 overlaps across environments for different testing set sizes (e.g., 112/0 and 52/0). From left to right, the NO/O ratio decreases with increasing overlapping genotypes. Vertical, black-dashed lines indicate PA for different reduced training sets (e.g., 112/0, 92/0, and 52/0).

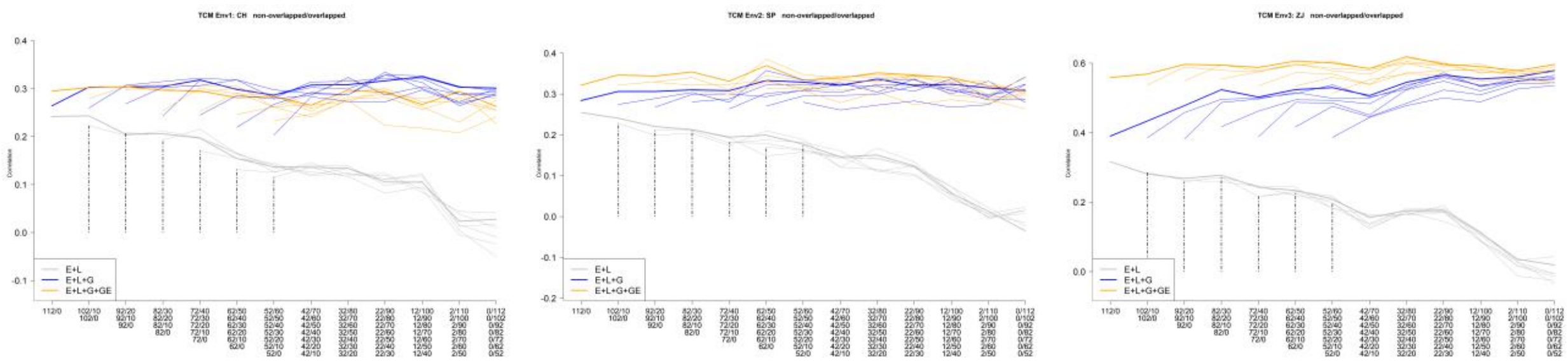

Figure S3 The correlation between predicted and observed values for varying TRAINING sets and allocation designs in TCM [Left: correlation for TCM in environment 1, Middle: correlation for TCM in environment 2, Right: correlation for TCM in environment 3] within each environment in three genomic prediction models, M1: **E+L;** M2: **E+L+G;** M3: **E+L+G+GE**. Bold lines show the average PA for the largest calibration set (112 genotypes). Dotted lines show the reduced training sets (102, 92, 82, 72, 62, and 52 genotypes). X axis shows the compositions of different non-overlapping (NO) / overlapping (O) allocation designs. Leftmost side of the X axis shows compositions for completely NO genotypes with 0 overlaps across environments for different testing set sizes (e.g., 112/0 and 52/0). From left to right, the NO/O ratio decreases with increasing overlapping genotypes. Vertical, black-dashed lines indicate PA for different reduced training sets (e.g., 112/0, 92/0, and 52/0).

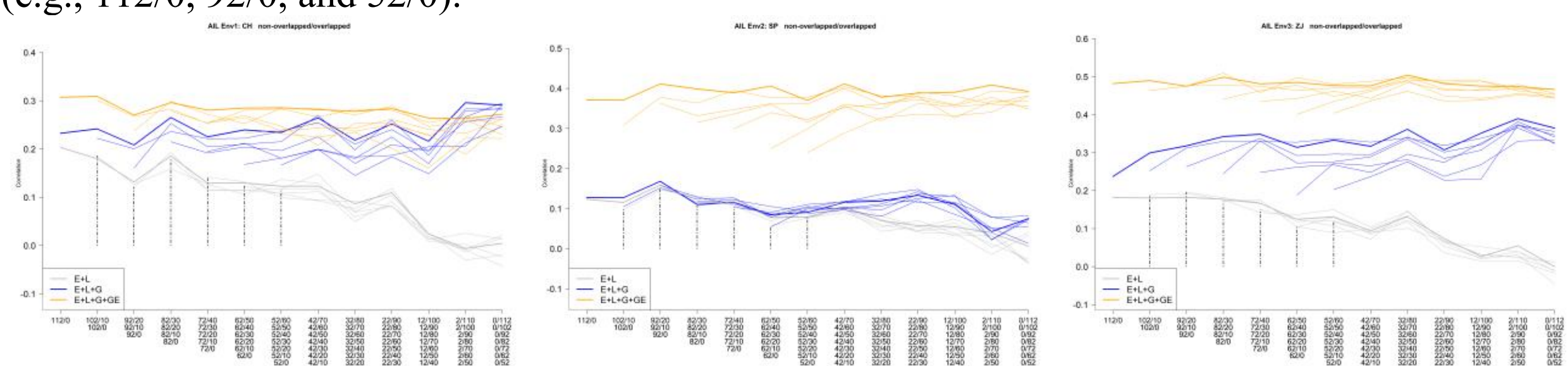

Figure S4 The correlation between predicted and observed values for varying TRAINING sets and allocation designs in AIL [Left: correlation for AIL in environment 1, Middle: correlation for AIL in environment 2, Right: correlation for AIL in environment 3] within each environment in three genomic prediction models, M1: **E+L;** M2: **E+L+G;** M3: **E+L+G+GE**. Bold lines show the average PA for the largest calibration set (112 genotypes). Dotted lines show the reduced training sets (102, 92, 82, 72, 62, and 52 genotypes). X axis shows the compositions of different non-overlapping (NO) / overlapping (O) allocation designs. Leftmost side of the X axis shows compositions for completely NO genotypes with 0 overlaps across environments for different testing set sizes (e.g., 112/0 and 52/0). From left to right, the NO/O ratio decreases with increasing overlapping genotypes. Vertical, black-dashed lines indicate PA for different reduced training sets (e.g., 112/0, 92/0, and 52/0).

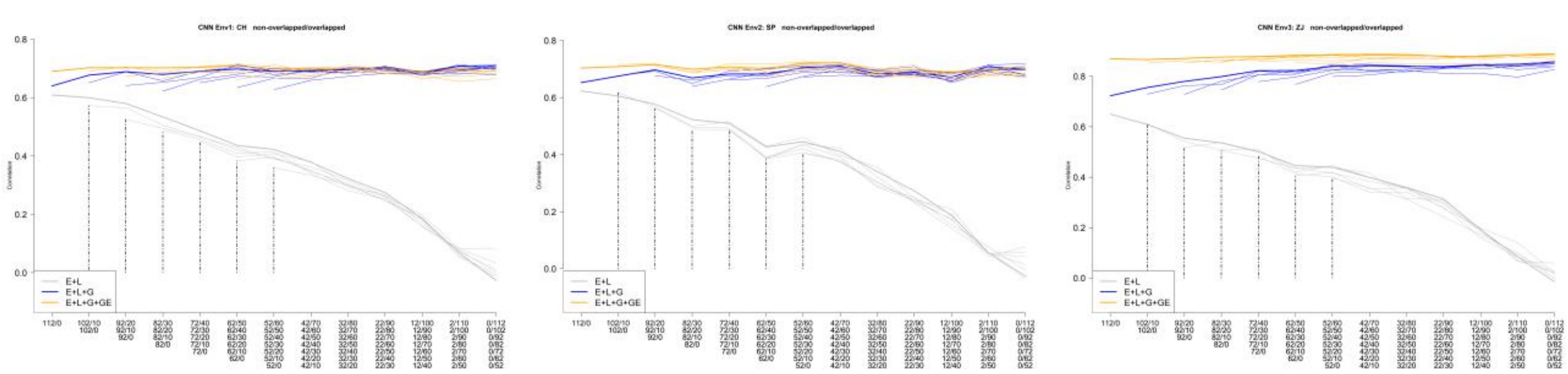

Figure S5 The correlation between predicted and observed values for varying TRAINING sets and allocation designs in CNN [Left: correlation for CNN in environment 1, Middle: correlation for CNN in environment 2, Right: correlation for CNN in environment 3] within each environment in three genomic prediction models, M1: **E+L;** M2: **E+L+G;** M3: **E+L+G+GE**. Bold lines show the average PA for the largest calibration set (112 genotypes). Dotted lines show the reduced training sets (102, 92, 82, 72, 62, and 52 genotypes). X axis shows the compositions of different non-overlapping (NO) / overlapping (O) allocation designs. Leftmost side of the X axis shows compositions for completely NO genotypes with 0 overlaps across environments for different testing set sizes (e.g., 112/0 and 52/0). From left to right, the NO/O ratio decreases with increasing overlapping genotypes. Vertical, black-dashed lines indicate PA for different reduced training sets (e.g., 112/0, 92/0, and 52/0).

Figures S6-S9
The MSE values for each trait within each environment.

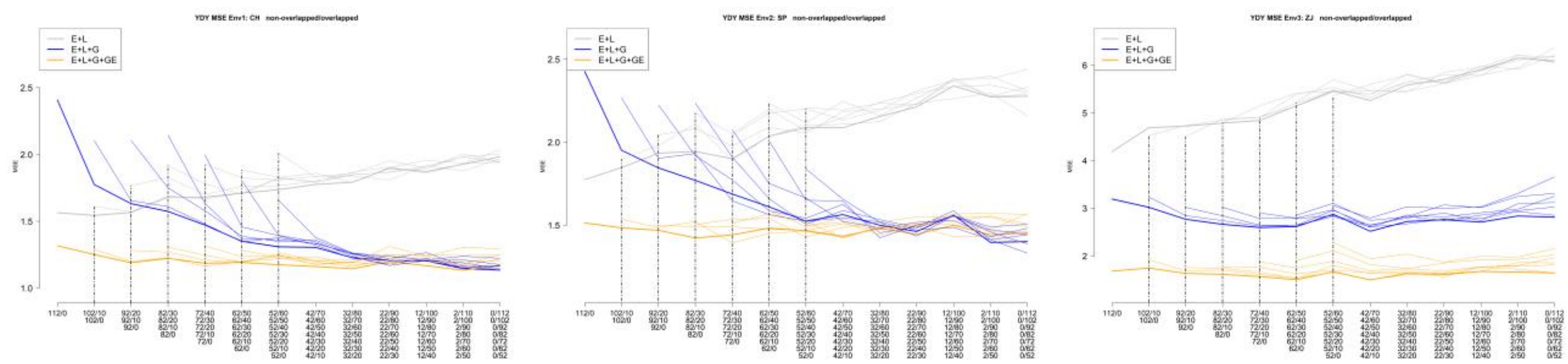

Figure S6 The MSE values for varying TRAINING sets and allocation designs in YDY [Left: MSE for YDY in environment 1, Middle: MSE for YDY in environment 2, Right: MSE for YDY in environment 3] within each environment in three genomic prediction models, M1: **E+L;** M2: **E+L+G;** M3: **E+L+G+GE**. Bold lines show the average PA for the largest calibration set (112 genotypes). Dotted lines show the reduced training sets (102, 92, 82, 72, 62, and 52 genotypes). X axis shows the compositions of different non-overlapping (NO) / overlapping (O) allocation designs. Leftmost side of the X axis shows compositions for completely NO genotypes with 0 overlaps across environments for different testing set sizes (e.g., 112/0 and 52/0). From left to right, the NO/O ratio decreases with increasing overlapping genotypes. Vertical, black-dashed lines indicate PA for different reduced training sets (e.g., 112/0, 92/0, and 52/0).

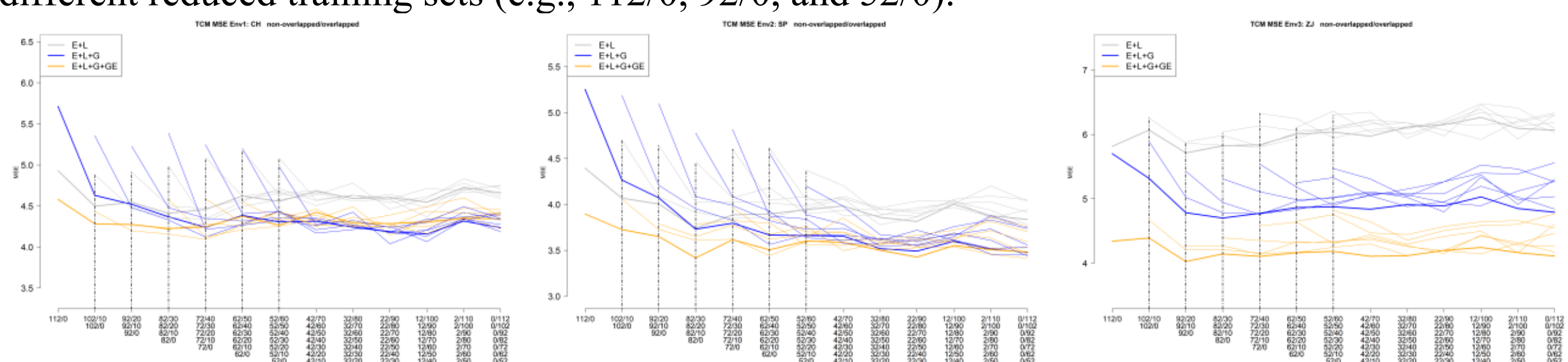

Figure S7 The MSE values for varying TRAINING sets and allocation designs in TCM [Left: MSE for TCM in environment 1, Middle: MSE for TCM in environment 2, Right: MSE for TCM in environment 3] within each environment in three genomic prediction models, M1: **E+L;** M2: **E+L+G;** M3: **E+L+G+GE**. Bold lines show the average PA for the largest calibration set (112 genotypes). Dotted lines show the reduced training sets (102, 92, 82, 72, 62, and 52 genotypes). X axis shows the compositions of different non-overlapping (NO) / overlapping (O) allocation designs. Leftmost side of the X axis shows compositions for completely NO genotypes with 0 overlaps across environments for different testing set sizes (e.g., 112/0 and 52/0). From left to right, the NO/O ratio

decreases with increasing overlapping genotypes. Vertical, black-dashed lines indicate PA for different reduced training sets (e.g., 112/0, 92/0, and 52/0).

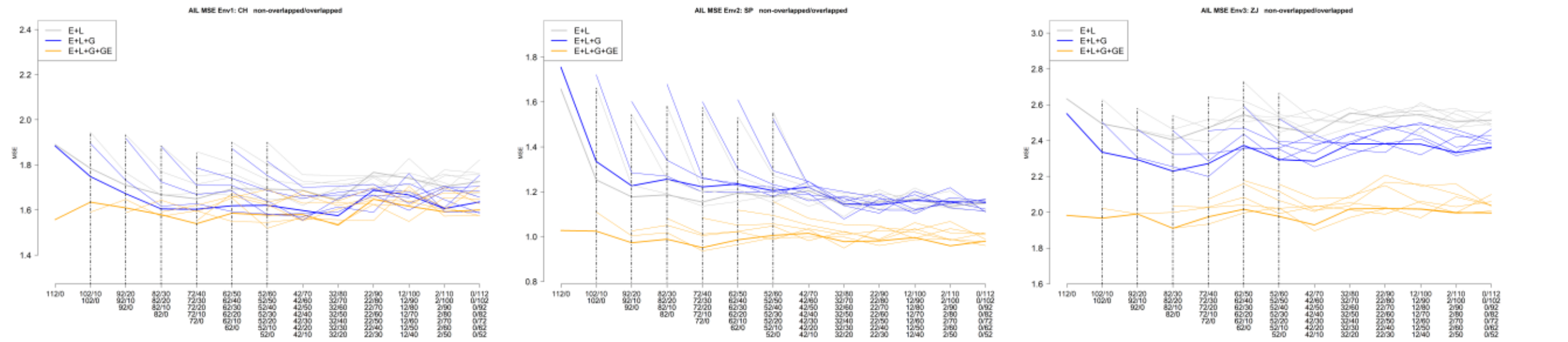

Figure S8 The MSE values for varying TRAINING sets and allocation designs in AIL [Left: MSE for AIL in environment 1, Middle: MSE for AIL in environment 2, Right: MSE for AIL in environment 3] within each environment in three genomic prediction models, M1: **E+L;** M2: **E+L+G;** M3: **E+L+G+GE**. Bold lines show the average PA for the largest calibration set (112 genotypes). Dotted lines show the reduced training sets (102, 92, 82, 72, 62, and 52 genotypes). X axis shows the compositions of different non-overlapping (NO) / overlapping (O) allocation designs. Leftmost side of the X axis shows compositions for completely NO genotypes with 0 overlaps across environments for different testing set sizes (e.g., 112/0 and 52/0). From left to right, the NO/O ratio decreases with increasing overlapping genotypes. Vertical, black-dashed lines indicate PA for different reduced training sets (e.g., 112/0, 92/0, and 52/0).

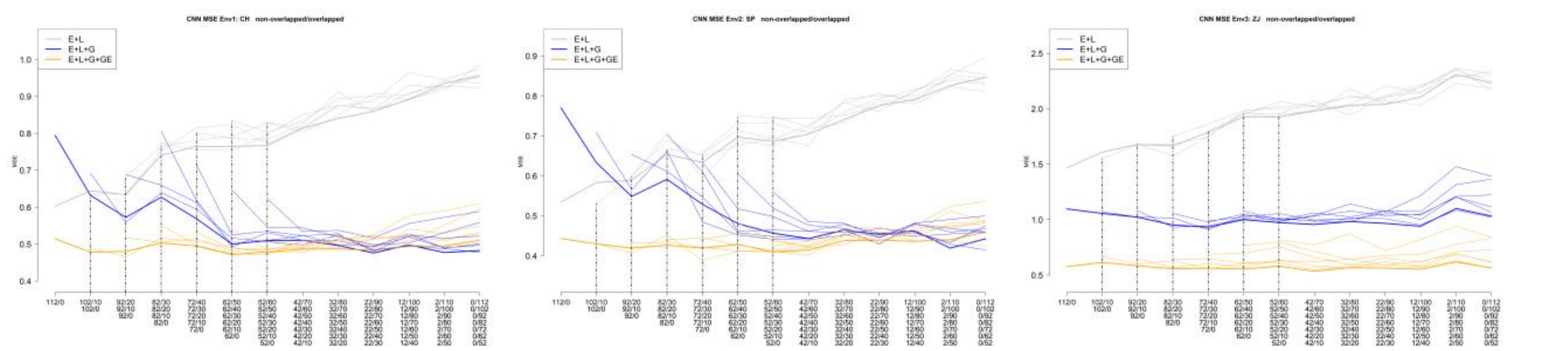

Figure S9 The MSE values for varying TRAINING sets and allocation designs in CNN [Left: MSE for CNN in environment 1, Middle: MSE for CNN in environment 2, Right: MSE for CNN in environment 3] within each environment in three genomic prediction models, M1: **E+L;** M2: **E+L+G;** M3: **E+L+G+GE**. Bold lines show the average PA for the largest calibration set (112 genotypes). Dotted lines show the reduced training sets (102, 92, 82, 72, 62, and 52 genotypes). X axis shows the compositions of different non-overlapping (NO) / overlapping (O) allocation designs. Leftmost side of the X axis shows compositions for completely NO genotypes with 0 overlaps across environments for different testing set sizes (e.g., 112/0 and 52/0). From left to right, the NO/O ratio decreases with increasing overlapping genotypes. Vertical, black-dashed lines indicate PA for different reduced training sets (e.g., 112/0, 92/0, and 52/0).
